# Resident and staff experiences of structural barriers to a housing-based overdose prevention site in Vancouver, Canada: “There is a double standard if you smoke”

**DOI:** 10.17269/s41997-025-01007-7

**Published:** 2025-03-18

**Authors:** Benjamin D. Scher, Benjamin W. Chrisinger, David K. Humphreys, Gillian W. Shorter

**Affiliations:** 1 Department of Social Policy and Intervention, University of Oxford, Oxford, UK; 2 Department of Community Health, Tufts University, Medford, MA USA; 3 Drug and Alcohol Research Network, School of Psychology, Queen’s University Belfast, Belfast, UK; 4 TreAdd Research Group On Treatment and Addiction, Tampere University, Tampere, Finland

**Keywords:** Housing-based overdose prevention site, Rapid ethnography, Harm reduction, Overdose crisis, Vancouver, Drug inhalation, Site de prévention d’overdose dans les logements, Éthnographie rapide, Réduction des risques, Crise d’overdose, Vancouver, Inhalation de drogues

## Abstract

**Objectives:**

Most overdoses in British Columbia (BC), Canada, occur within housing settings. In response, the provincial government is increasingly implementing housing-based overdose prevention sites (HOPS). Within the context of a contaminated drug supply, and changing consumption practices, there is little research examining the effectiveness of HOPS. The aim of this study was to explore qualitatively how residents and staff experience HOPS, focusing on how this intervention fits into the day-to-day operations of a low-barrier housing facility.

**Methods:**

This study was undertaken at a non-profit housing and emergency shelter facility, with a HOPS in Vancouver, BC. We employed rapid-ethnographic methods including six weeks of non-participant observation (> 200 h), three focus groups, 20 informal interviews with residents, and 10 semi-structured interviews with staff. Data were analyzed through an inductive thematic approach.

**Results:**

Our results suggest that this facility’s HOPS is underutilized due to a variety of structural factors, the most prominent of these being the lack of inhalation services. This lack of service provision exacerbates overdose vulnerability and stigma. Continued drug consumption near the building and in non-monitored areas inside the building creates challenges for staff in identifying potential overdoses and exposes residents who do not consume drugs to drug use within the building.

**Conclusion:**

Housing provision which provides a safer consumption environment to include those who smoke drugs is urgently needed to support both individuals who smoke and those looking to transition from injecting to smoking.

**Supplementary Information:**

The online version contains supplementary material available at 10.17269/s41997-025-01007-7.

## Résumé

**Objectifs:**

La plupart des overdoses en Colombie-Britannique (BC), au Canada, se produisent dans des logements. En réponse à cette situation, le gouvernement provincial met de plus en plus en place des sites de prévention d’overdose dans les logements (HOPS). Dans le contexte de la contamination de l’approvisionnement en drogues et de l’évolution des pratiques de consommation, peu de recherches ont été menées sur l’efficacité des HOPS. L’objectif de cette étude était d’explorer qualitativement les façons dont les résidents et les employés experiencent les HOPS, en se concentrant particulièrement sur la façon dont cette intervention s’intègre dans les activités quotidiennes d’un centre de logement avec un HOPS.

**Méthodes:**

Cette étude a été menée dans un centre de logement et d’hébergement d’urgence avec un HOPS à Vancouver, BC. Nous avons utilisé des méthodes d’ethnographie rapide, notamment six semaines d’observation non-participante (>200 heures), trois groupes de discussion, 20 interviews informels avec des résidents et 10 interviews semi-structurés avec des employés. Une approche thématique inductive a été utilisée pour analyser les données générées.

**Résultats:**

Nos résultats suggèrent que l’HOPS de cet établissement est sous-utilisé en raison de divers facteurs structurels, le plus important étant l’absence de services d’inhalation. Ce manque de services crée la vulnérabilité aux overdoses et la stigmatisation. La consommation de drogues continue à proximité du bâtiment et dans des zones non surveillées à l’intérieur du bâtiment. Cela pose des problèmes aux employés pour identifier les overdoses potentielles et expose les résidents qui ne consomment pas de drogues à la consommation de drogues à l’intérieur du bâtiment.

**Conclusion:**

Il est urgent de créer et mettre en place des logements qui offrent un environnement de consommation plus sûr pour les fumeurs de drogues, afin de soutenir les fumeurs et les personnes qui souhaitent changer leur mode de consommation de l’injection à l’inhalation.

## Introduction

Canada continues to face an unyielding overdose crisis. There have been 47,162 apparent opioid toxicity deaths reported between January 2016 and March 2024 and drug deaths have now surpassed all other forms of accidental death combined (Public Health Agency of Canada, 2024). This overdose crisis has largely been driven by a shift in the drug supply, marked by the proliferation of synthetic opioids such as fentanyl, and many other synthetic analogues at unexpected strengths (Pardo, 2021).

On April 14, 2016, British Columbia (BC) declared a public health emergency. To better respond to rapidly escalating numbers of drug-related deaths, this declaration provided the legislative and financial support required to expand the delivery of harm reduction services (Fischer et al., 2019). Although primarily implemented in Vancouver, such interventions include safe supply programs, opioid agonist treatment (OAT), expanded naloxone distribution policies and programs, drug checking services, and various models of overdose prevention sites including housing-based overdose prevention sites (HOPS) (Strike & Watson, 2019). Although Canadian service coverage remains limited (they exist predominantly in BC), HOPS are designated spaces within housing facilities where residents’ drug use (typically limited to injecting and snorting) is supervised by either peers or other members of staff who can respond in an overdose event (Bardwell et al., 2019).

Despite these innovations, BC remains the Canadian province most severely affected by drug-related deaths. In 2023, BC recorded 2511 drug-related deaths, the largest number on record (BC Coroners Service, 2024). Fentanyl was detected in 85% of these deaths, with most occurring when people smoked their drugs (71%), followed by nasal insufflation (15%), injection (12%), and oral consumption (6%) (BC Coroners Service, 2024). Over time, methods of drug consumption and the mode of consumption associated with overdose have shifted. Before 2016, most drug-related deaths in BC resulted from injection (Parent et al., 2021). Qualitative studies have revealed that peoples’ motivation for switching from injecting to smoking (primarily fentanyl) includes difficulties accessing veins, a desire to reduce the risks associated with injecting, fewer financial constraints, reduced stigma, increased pleasure, and more perceived control over the intensity of effect (Harris et al., 2020; Kral et al., 2021; Pijl et al., 2021).

Evidence both globally and BC-specific highlights the structural and environmental links between housing and overdose risk (Bardwell et al., 2019; Collins et al., 2020; Ivsins et al., 2022; Milaney et al., 2021; Norton et al., 2022). A primary risk factor is people using alone in their rooms. This behaviour is driven by several contextual and structural barriers, including a desire for privacy, a perceived safer alternative to other available consumption environments, and exclusion from the available supervised consumption environments (Fleming et al., 2024a, 2024b; Papamihali et al., 2020; Winiker et al., 2020). In 2024, 83% of drug deaths in BC occured inside (48% in private residences and 35% in other residences, including social and supportive housing, single room occupancies, shelters, hotels, and other indoor locations) and 16% occurred elsewhere (e.g., vehicles, sidewalks, streets, and parks) (BC Coroners Service, 2024). In response, an increasing number of housing facilities for people who use drugs have integrated HOPS.

A growing body of public health research has examined housing interventions that reduce overdose vulnerability (Bardwell et al., 2019; Collins et al., 2020; Fleming et al., 2024a, 2024b; Ivsins et al., 2022; Milaney et al., 2021; Wallace et al., 2018). Despite this, no research has focused explicitly on HOPS in facilities housing residents who use drugs and residents who do not, or on the perspectives of staff within these facilities. Our study sought to respond to this research gap by studying a mixed housing setting and incorporating the views of staff to get a holistic understanding of the operational context of HOPS and their host housing facilities.

## Methods

### Study setting

The setting for this study was a housing facility with a HOPS in Vancouver, BC, outside of the Downtown Eastside. This housing facility incorporated three separate resident programs. The first was an emergency shelter program, with people living in shared dorms in the basement. The second was a temporary accommodation program where residents room on the ground and first floor for up to 3 months. Finally, the third program on floors 2–4 was for long-term tenants who had provincial social housing. All residents, regardless of which program they were a part of, were assigned case workers to help resolve their housing insecurity and support them in accessing other health, social, welfare, and legal services (e.g., employment assistance, primary care, mental health support, drug treatment, applying for ID or social welfare assistance). All services were provided on-site or through referral to services in the local neighbourhood. Meals, laundry, medication dispensing, storage, and a common room with a TV, video games, and computers were available on-site. All residents could access harm reduction supplies from the front desk, as well as the HOPS next to the front desk which has been in operation since November 2018.

HOPS vary in design and operational context (e.g., staff or peer monitored, self-initiated monitoring, virtual monitoring with cameras) (Collins et al., 2020). Here, when we refer to HOPS, we mean a designated monitored space, accessible to all residents for injecting or snorting drugs. This specific HOPS was a small room connected by two doors, one to the reception for staff access and one leading to the common room for residents. Residents needed to check in with staff and disclose what they intended to consume before they could enter. The staff entrance door had a window allowing staff to check on residents. Staff set a re-occurring 2-min timed alarm at their desk once someone had entered as a reminder to conduct a check. The room comprised a stainless-steel desk with a mirror in front. Around the walls were colourful shelves with harm reduction supplies, pieces of artwork, and posters with harm reduction advice. Only one person could use the room at a time and there was no time limit for use. Staff encouraged residents to relax in the common room post consumption as a monitoring strategy. Naloxone and oxygen were available to respond to an overdose.

### Data generation

Between March and April 2023, we conducted six weeks of rapid ethnography, a conceptual and methodological approach which allows for the generation of quick, in-depth insights and timely recommendations regarding policy and practice based on lived experience (Vindrola-Padros, 2021). Given the relatively short timeframe of fieldwork, qualitative methods were employed with the specific aim of rapidly, yet accurately, capturing and describing how the HOPS and wider operational policies of the facility were perceived and experienced by residents and staff. This was a pragmatic approach to evaluate the key features of the service (Vindrola-Padros & Vindrola-Padros, 2018) and the strengths and weaknesses of the current delivery. Methods of data collection included (1) non-participant observation, (2) a community consultation, (3) focus groups with residents, (4) ethnographic interviews with residents, and (5) semi-structured interviews with staff (including reception staff, caseworkers, and management).

### Non-participant observation

The lead author (BDS) completed approximately 200 h of non-participant observation where between 9 am and 5 pm on weekdays, he shadowed staff during intakes and case planning sessions, and spent time with residents during mealtimes and in the common room. Although he could not be in the HOPS with clients, he spoke with them before and after they used the room. This initial period enabled him to build rapport with residents and staff prior to the focus groups and interviews. It was not possible to get consent from every person present during the non-participant observation period; therefore, field notes collected were generalized and did not refer to specific residents or events.

### Ethnographic interviews with residents

During this period, BDS conducted 20 informal, one-on-one ethnographic interviews (see Supplementary Material, Appendix 3, for protocol). Handwritten notes captured key quotes which were subsequently read back to participants to check for accuracy. The total informal ethnographic interview participant group included 16 men, 3 women, and 1 transgender woman, 15 of whom self-identified as white, 3 as Black, and 2 as First Nations/Métis/Inuit. Prior to each interview, the study was explained and written consent was gained. These conversations also presented the opportunity to inform interviewees about the focus groups which would take place at a later stage.

### Focus groups

In the final two weeks of fieldwork, three five-participant focus group discussions (*n* = 15 participants) were conducted and facilitated by BDS. The total focus group participant group included 12 men, 2 women, and 1 transgender woman, 11 of whom self-identified as white, 3 as Black, and 1 as First Nations/Métis/Inuit. Ages ranged between 34 and 60 years of age. Focus groups were audio recorded and subsequently transcribed. Recruitment was done through purposive sampling, with most participants identified from built rapport during the earlier fieldwork stage. Focus groups ranged between 29 and 44 min in duration. A focus group protocol (see Supplementary Material, Appendix 2) of eight open-ended questions covered residents’ daily experiences in the facility and with the HOPS. Residents who used drugs and those who did not were invited to partake in the focus groups to attain a holistic and representative sample of client experiences. We did not collect demographic data in relation to who used the HOPS; however, over the course of the fieldwork, there were only five residents who used the room and they all took part in both the interviews and focus groups.

### Staff interviews

Ten one-on-one interviews were conducted with staff (see Supplementary Material, Appendix 4, for protocol). We were only permitted to interview staff during their breaks, which restricted interview duration to between 5 and 17 min. Questions were oriented towards understanding staff perspectives of the day-to-day operations of the facility and the HOPS.

### Ethics and ethical considerations

Staff were not compensated for their participation; however, focus group participants were compensated CAD$15 and resident interviewees were compensated CAD$10. To protect anonymity, age and ethnicity were not recorded for the staff interviews and age was not recorded for the resident interviews. This project received ethical approval from the University of Oxford Central Research Ethics Committee (REF: R84228/RE004) and the board of the host organization (not named to ensure anonymity). Residents also verified and commented on the protocol during a community consultation which took part in the fourth week of fieldwork. See COREQ checklist (Supplementary Material, Appendix 1) for further details on method and researcher positionality (Tong et al., 2007). To ensure privacy and anonymity, each interview and focus group was conducted in a private room. Recognizing some individuals may not feel comfortable speaking freely during an interview located in the service they are being asked to reflect on (Hameed et al., 2018), we attempted to mitigate potential bias by reassuring participants their responses would be anonymous.

### Data analysis

Focus group and staff interview transcripts, resident interview notes, and non-participant observation fieldnotes were imported into NVivo Software and coded by BDS using six steps of inductive thematic analysis (Braun & Clarke, 2006). This process involved (1) BDS reading transcripts and fieldnotes for the purpose of data familiarization; (2) BDS writing analytic memos and developing initial codes (29 from resident interviews, 59 from focus groups, and 26 from staff interviews); (3) BDS developing a thematic codebook to work systematically through the data, identifying relevant, meaningful information related to the research questions; (4) all authors reviewing, collaboratively defining, and finalizing the themes; and (5) BDS writing the first draft of the manuscript, with review and editing by all authors. Although informed by our understanding of the Risk Environment Framework (Rhodes, 2002) and how environmental, social, and policy factors produce risk within housing environments (Braubach & Fairburn, 2010; Ivsins et al., 2022), all codes were developed inductively from the data.

## Results

Three central themes were derived from the narratives, experiences, and perspectives of residents and staff: (1) structural barriers to HOPS accessibility, (2) experiences of residents and staff around the building, (3) experiences of residents and staff within the building.

### Structural barriers to HOPS accessibility

Residents and staff described several structural factors which directly influenced people’s willingness and ability to access the HOPS. These included (1) the stigma of accessing the room and the fear of it affecting treatment by staff, (2) restrictions on HOPS capacity and consumption practices, and (3) exclusion of people who smoke their drugs.

#### Stigma of accessing the room and the fear of it affecting treatment by staff

Residents repeatedly perceived stigma from staff associated with those who used the HOPS. This produced anxiety, primarily stemming from the belief that by accessing the room and in turn revealing their drug use, staff would view and treat them differently from the residents who did not use drugs:

Fear of judgment, you still have to admit it out loud and that is a huge step for a lot of people. So I think people still believe that if they use the room staff will treat them differently or put something in their file. (Resident Interview 17, white, trans-female)

The notation on “their file” reflected a fear this could cause a formalized, permanent marker of their drug use and a way to monitor their drug use. Certain staff corroborated this sentiment, noting that despite the HOPS, residents were still worried about being stigmatized around their drug use and particularly in relation to the amount used. Their fears had foundation as this staff member stated:

They don’t want to be stigmatized. They think they’re being judged...And on some level I think there’s some truth to that. They don’t want us knowing how much they use. (Staff Interview 2)

Fieldnotes from observations documented how staff explained HOPS policies and procedures during resident intakes in a clear, neutral manner and encouraged service access. However, new residents would have to feel comfortable disclosing their drug use during the initial intake, often before rapport with staff had been built. Understandably, new residents did not always feel comfortable doing this.

#### Restrictions on HOPS capacity and consumption practices

The rules of the HOPS were oriented around managing capacity, accounting for staffing numbers, and abiding by broader provincial policies governing such harm reduction services. One key rule was that only one person could use the HOPS at a time. This had a profound impact on couples who managed and supported each other’s consumption practices:

They should have a couples [HOPS] room…it just sucks to be split up when we could be together outside. We still sometimes use outside or we go to an OPS where they do let us use together, but that is back in the Downtown Eastside. (Resident Interview 6, white, female)

By not accommodating social dynamics surrounding people’s drug use, couples described being pushed to use outside the building or having to commute far from their new residence to stay together. Many others used in pairs or in groups to mitigate health risks or manage existing physical health issues. In this focus group, participants describe why this can be problematic:

- Someone should be able to do the needle for you, to doctor, it’s very important. (FG Participant 14, white, male)
- I’m usually the one that cooks it and helps her. (FG Participant 13, white, male)
- And you aren’t able to help her? (Moderator)
- No, they won’t listen or let us in together. (FG Participant 13, white, male)
- The thing is [I] have that problem [as well]. It’s serious, I can’t do it [inject] myself because I can’t see that well. (FG Participant 15, Indigenous, male)
- You end up wasting it or having to get more. (FG Participant 13, white, male)
- Or poking away and having to heal a whole bunch of places you missed. Then it brings infection and…you gotta go to the hospital. (FG Participant 14, white, male)

Not being able to use as a pair or to help one another, something people described doing in other consumption settings, resulted in people either losing/not maximizing their dose, or experiencing injecting-related harms and increased chance of hospitalization.

#### Exclusion of people who smoke their drugs

Noted both in the fieldnotes and in resident testimonies, there were residents who injected drugs for whom the HOPS was accessible and was effectively reducing their risk of fatal overdose:

I think it’s a good thing because I dropped yesterday and they were really fast on getting me back up. (FG Participant 15, Indigenous, male)

Perspectives of residents who did not use drugs also reflected the increased safety to people who inject drugs:

I don’t use the [HOPS] room, it doesn’t bother me but I am glad they have a safe place to go. It must be scary on the street. (Resident Interview 3, white, male)

I don’t use but I am glad that they have it for the safety of those who do use. (Resident Interview 15, white, female)

There was also a recognition that “the larger percentage of people here…don’t use needles” (FG Participant 11, white, male). Participants perceived a fundamental gap in service provision of the HOPS as it had no facilities to support people who smoke their drugs or those seeking to transition from injecting to smoking. Staff understood this and regularly encouraged people to access alternative supervised smoking sites:

So in this facility no, but we do strongly advise them to go down to the [different OPS facility which does permit smoking], where they do have an open-air space where they can actually smoke their drugs and be monitored in the same way. (Staff Interview 6)

As fieldwork, focus groups, and interviews progressed, the lack of smoking provision was repeatedly communicated as a major barrier to people accessing the HOPS. This was acknowledged by residents who made comments such as “there must be a way to offer people a safe place to smoke as well” (Resident Interview 20, Indigenous, male). This finding is reflected in the subsequent themes.

### Experiences of residents and staff around the building

The HOPS did not possess the required ventilation systems or policy framework to support people who smoked drugs. This resulted in people being pushed into more marginal settings, often either outside of the entrance on the front steps, or in the alley way behind the facility. This increased risks and harms for residents and posed challenges for staff trying to keep residents safe.

#### Navigating risks and harms smoking drugs around the building

By using outside, residents placed themselves in an environment which produced intense and real feelings of self-stigma. Here, this participant adds how attempts to switch from injecting to smoking were complicated because of the absence of a safe space for smoking:

As an opiate user there is a lot of stigma. I’ve been using drugs since I was 8 years old and…I am trying to stop with the needles but there is no safe space for me to smoke. Now I have to stand out hiding on the corner by the cops and that does make me feel like a junkie. It makes being here very complicated. (Resident Interview 10, white, male)

This participant re-affirms the discrepancy in the harm reduction options available to those who inject and those who smoke by describing how those who inject can mitigate overdose risk and shield from the elements, whereas residents who smoke have no other option but to use in unsafe, exposed environments:

I don’t use needles and do think it is a good thing but then again there is a double standard. If you use needles you have a safe space but if you smoke your dope like me then you are out in the fucking cold or you get kicked out. Like now I am still using in the alleys in front of people when I’m supposed to be getting away from all of that. But if I decided to inject now I would be fine. (Resident Interview 9, Indigenous, male)

Participants described using outside as a risk factor for overdose, irrespective of their housing status:

I’ve lost 30 people in the last year and I don’t want to become another statistic but I’m still having to use in an alley despite now having this housing. (Interview 10, white, male)

Together, these quotes highlight the limitations of an injection-only HOPS, and the increased risk of harms and overdose for residents who smoke versus those who inject their drugs.

#### Staff challenges in responding around the building

Both ethnographic fieldnotes and staff accounts documented how staff attempted to monitor use outside. When staff knew a resident was going outside to smoke drugs, they encouraged use in a location monitored by the cameras. This acted as an informal supervised consumption environment and allowed staff to intervene if an overdose occurred, allowing them to recognize the overdose, call an ambulance, and administer naloxone. This was a compromise, one which was not ideal for staff or residents:

They have to go outside, and the problem is that we ask them to use around the corner so that they’re on camera, but people don’t want to be on camera. (Staff Interview 2)

Residents or members of the public would come into the building to alert front-desk staff if someone was overdosing near the building. Staff had a rota for conducting perimeter checks of the building to increase their chance of intervening in the case of an overdose. Despite this, overdose was a common occurrence:

Yeah, people have gone down all around this building from smoking. (Staff Interview 10)

Overdoses near the building were common and attributed to people smoking their drugs. As alluded to in this previous quote, instances of overdose were not confined to outside the building.

### Experiences of residents and staff in the building

As a result of perceived or actual barriers discussed in the first theme, people sought environments within the building to consume drugs—particularly for smoking. This was an important issue for residents who did not use drugs who witnessed drug use at their home, and by staff who worked to keep people safe.

#### Client experiences and challenges

Drug use outside of the HOPS but in the building was commonly described and observed: “Loads of people are still using in the washroom” (Resident Interview 17, white, trans-female). Participants described logical reasons for seeking such alternative locations: “It’s…pissing it down [raining] out…who wants to go out there to use? Come on.” (FG Participant 11, white, male). In addition to avoiding bad weather and the harms associated with preparing and consuming drugs in those conditions, participants also described how navigating such risks had to be balanced against the tangible risk of losing their space with the accommodation provider. This interaction between participants speaks to this risk:

- So I know I’m not allowed to use…you’re not allowed to smoke drugs down in the bathrooms…but you know there are going to be people using the safe injection [room]…I don’t use needles so I don’t use that room…[but] if I got caught using downstairs…I would risk my bed here. (FG Participant 11, white, male)
- Even though there is a bathroom down there and that is a very risky thing to do…because I’m risking my stay, right, but at the same time you don’t care because it’s raining outside. (FG Participant 14, white, male)

Consuming drugs in a locked, inaccessible washroom brings additional risks beyond overdose. This participant describes an instance in which the smoke from someone consuming drugs set off the fire alarm:

Somebody was smoking crystal meth two weeks ago in the bottom bathroom…someone went down and…two minutes later the fire alarm was pulled. Then somebody upstairs was doing the same…and I started to wonder, it’s not smoke [from a fire] that’s causing the fire alarm, it’s…somebody doing crystal…or…crack [which] is causing the fire alarm [to go off]. (FG Participant 9, white, male)

The decision by some residents to seek out alternative drug consumption locations again highlights the need for interventions that support residents who smoke drugs. By reducing overdose risk, minimizing the visibility of drug use within the building, and addressing secondary issues such as the likelihood of false fire alarms, these interventions would benefit all who live in the facility, regardless of whether they smoke drugs.

#### Staff challenges responding to smoking within the building

Staff described the challenges smoking in the building presented for them in their day-to-day work. They also referenced the key deterrent strategy which was a zero-tolerance policy for drug use inside the building, resulting in eviction if caught:

Multiple times here…this person came in was told hey, do not use or you’ll be kicked out and what ends up happening? We find drug paraphernalia...laid out on his bed, ready to use or he’s laid out with a pipe on his chest. (Staff Interview 4)

Dorms were shared between people who use drugs and people who do not or who had recently begun drug treatment. A key concern was triggering people who were newly abstinent into potential relapse.

It is an issue…especially in the downstairs bunk area, because we have a mix of people who are trying to get clean and people who are still deep in their addictions…and so you got people...using in the bathroom at 2–3 in the morning and some who [are] trying to get off. (Staff Interview 4)

Staff concerns were often centered on overdose risk and hidden drug use amounting to overdose events in which they could not intervene. Staff expressed that “mostly the biggest challenge is keeping people safe” (Staff Interview 7), suggesting that the current provision may not keep both residents who use and those who do not use safe. Despite the HOPS being used by residents who injected drugs, responding to overdose or having to enforce rules around use inside the building continued to take up a substantial amount of staff time.

## Discussion

As provincial and federal governments seek to refine harm reduction provision and respond to trends in local drug consumption practices, it is helpful to understand gaps in the design and delivery of existing services. The experiences and perspectives of participants in this study highlight some structural barriers around smoking in this facility, likely replicated in other sites in BC and North America, where there is a shift from injecting to smoking. This shift is often in response to the risks associated with injecting and is demonstrative of people attempting to reduce their own personal health risks (Harris et al., 2020). This primary finding reinforces the recognition that emphasizing only individual behaviour change cannot alone reduce intersectional risk (Collins et al., 2020).

Being or feeling surveyed within the context of HOPS usage, or being supervised through cameras outside of the building, may dissuade some from accessing the current harm reduction intervention on offer. Existing research highlights the myriad forms of surveillance found across harm reduction programs, such as disclosure at intake, ongoing check-ins with staff, and monitoring post consumption (Scher, 2020). In this context, the feeling of surveillance may be alleviated by employing peer workers to directly supervise HOPS clients. Research examining facilitators to community-based overdose prevention services suggests that peer-run services where the frontline staff are not directly involved in the delivery of auxiliary services such as case management may help residents feel more comfortable and more likely to attend (Ivsins et al., 2023). Structural barriers, including those prohibiting people from using together or sharing their drugs, dissuaded people from accessing overdose prevention services in community settings. Rhodes et al. (2017) note people may want to use together to overcome physical impairment or difficulties accessing veins, although it is not without risks, including around relational dynamics, even when in a safe, secure, and supervised environment (Boyd et al., 2018; Collins et al., 2019; Pijl et al., 2023).

Although the HOPS was being accessed by residents who injected drugs—i.e., their risk was not increased by the environment—risk and harm were produced through the lack of smoking provision and led to the HOPS being under-used. Most residents resided in shared dorms and did not have their own rooms they could lock. Therefore, most of the drug consumption happened outside of the building and in washrooms, increasing the risk of overdose and jeopardizing safety. Safety, trust, and inclusion are key mechanisms for ensuring consumption spaces are effective (Stevens et al., 2024); staff, constrained by space and by operational requirements, are unable to fully safeguard clients from harms within their residential risk environment. Residents reported stress and anxiety associated with drug use in the absence of a safe space where they could smoke. Building on the findings of Collins et al. (2020), our findings highlight how the “privileging of injection drug use in HOPS can reinforce fatal overdose vulnerability” (p. 7). While infrastructure change may be required, there are a range of solutions, piloted in other contexts, that could be considered to support this population and their desire to minimize risks (Shorter et al., 2023).

Staff and residents who do not use drugs were also impacted by people being pushed into consumption environments inside and outside of the building. Previous research has suggested that witnessing drug use in a shelter setting can act as a trigger for relapse (Binswanger et al., 2012). Safety, for all residents, encompasses interpersonal and environmental factors, in addition to overdose or relapse risks. Ensuring a safe environment for all residents is crucial; however, staff and management are often constrained by operational policies and resources at the micro and macro levels (Collins et al., 2019; Kerman et al., 2023). For this reason, it is essential that federal and provincial leadership support the needs of all residents in such facilities, while acknowledging the pressing overdose context.

People are at significantly more risk of overdose when not housed (Milaney et al., 2021). As such, solutions are imperative to reduce the likelihood of people using drugs in unsafe environments or even dropping out of housing programs due to the inability of services to cater for their consumption practices (Kerman et al., 2023). In this facility, there was already some provision for people who smoke drugs through safe smoking supplies dispensed at the front desk. This however did not reduce the risks associated with their immediate consumption environment. The influence of the environment on risks affecting people who use drugs is widely known (Collins et al., 2019; Rhodes et al., 2017), and this study echoes risks highlighted in other housing settings where HOPS have not been implemented.

Inhalation provision is common in supervised consumption settings in Europe (Bourque et al., 2019) and provides example models for adoption in Canadian HOPS settings (Shorter et al., 2023). There would be considerable benefit to adapt HOPS to support residents who smoke. Ideally, they would have capacity for a minimum of two people at a time to account for important social dynamics pertaining to people’s drug use. Peers may also provide crucial support in monitoring, preventing, and intervening in overdose situations. Employing peers would also reduce the workload for existing staff (Kennedy et al., 2019), who are under considerable pressure managing current macro and micro risks at the HOPS. Effective “inclusion health interventions” that can evolve to meet the diverse health and social needs of clients in addition to managing overdose risk (Scher et al., 2024) can bring benefits for staff, residents, and communities alike. Building purpose-made ventilated rooms alongside current injecting rooms, although ideal, may not be feasible. Instead, an adaptation could take the form of a supervised inhalation tent in a secure outside area such as a courtyard or garden (Borque et al., 2019; Pijl et al., 2023).

### Limitations

Given that some of the ethnographic interviews were only 5 min in length, there is a chance that critical aspects of participant experiences in the service may not have been conveyed in depth. Similarly, aspects of intersectional risk environments (e.g., poverty, race, gender, and stigma) may have been underrepresented. Although resident views were triangulated with those expressed during the focus groups, staff views may be incomplete given these interviews were the sole source of data collection. As such, while the rapid nature of this study allowed us to quickly capture rich data and make timely recommendations within an urgent policy context, a longer-form ethnography may still be warranted. Additionally, the study was conducted within a single housing setting in Vancouver, BC, which limits the generalizability of the findings to other settings or regions. Conducting the interviews and focus groups within the setting of interest may also have influenced participant responses; however, by assuring anonymity, we hoped to mitigate this influence. Finally, this study did not quantitively measure the rates of use of the HOPS. It would have been useful to capture its uptake alongside participant experiences and perspectives.

## Conclusion

Seeking to respond to a gap in the public health literature, our qualitative study captures the experiences and perspectives of residents—both those who use and those who do not use drugs—and of staff within a supportive housing facility with a HOPS in Vancouver, BC. Although residents who injected drugs benefited from the harm reduction outcomes of the HOPS, particularly safeguarding from fatal overdose, our findings suggest this HOPS is underutilized due to several structural factors, the most prominent being the lack of inhalation services. This lack of service provision and the creation of an environment in which people conceal their drug use shape several harms for both residents who use drugs and those who do not. These additionally create challenges for staff responding to drug consumption near the building and in non-monitored areas inside the building. As such, there is an urgent need for policymakers to consider changing modes of drug consumption within existing housing-based harm reduction interventions and implementing broader policy responses to combat the ongoing overdose crisis.

## Contributions to knowledge

What does this study add to existing knowledge?

- This study has uncovered several structural barriers that impede access to a housing-based overdose prevention site in Vancouver, Canada; most notably, the lack of inhalation service provision.
- The lack of smoking provision shapes several harms for both residents who use drugs (e.g., increased risk of isolated drug use) and those who do not (e.g., exposure to drug use in their immediate living space).
- The lack of smoking provision creates significant challenges for staff responding to drug consumption near the building and in non-monitored areas inside the building.

What are the key implications for public health interventions, practice, or policy?

- Housing services must urgently adapt their harm reduction strategies to address the risks faced by residents who smoke drugs.
- Policymakers need to consider changing modes of drug consumption within existing housing-based harm reduction interventions and implementing broader policy responses to combat the ongoing overdose crisis.

## Supplementary Information

Below is the link to the electronic supplementary material.

Supplementary file1 (DOCX 35 KB)

## Data Availability

Data available upon reasonable request.

## Code availability

N/A.
