# Supplementary material for: Resident and staff experiences of structural barriers to a housing-based overdose prevention site in Vancouver, Canada: “There is a double standard if you smoke”
Source: Can J Public Health. 2025 Mar 18;116(3):387–97. doi: 10.17269/s41997-025-01007-7 (PMC12381312; doi:10.17269/s41997-025-01007-7)
Supplement: Supplementary file 1 — Supplementary file1 (DOCX 35 KB) [file 41997_2025_1007_MOESM1_ESM.docx]

**Title**: Resident and Staff Experiences of Structural Barriers to a Housing-Based Overdose Prevention Site in Vancouver, Canada: “There is a double standard if you smoke”

**Appendix 1:** Consolidated criteria for reporting qualitative studies (COREQ): 32-item checklist (items 1-5 relate to positionality and personal characteristics of the authors and can be included in the final table after the peer-review process to maintain anonymity)

| No | Item | Guide Questions/Description |  |
| --- | --- | --- | --- |
| Domain 1: Research Team and Reflexivity |  |  |  |
| Relationship With Participants |  |  |  |
| 6. | Relationship established | Was a relationship established prior to study commencement? | Yes, the author had worked and conducted research for and with this organization for two years, three years prior to this study and respective fieldwork taking place. This included working at the site involved in this study. He also conducted his MA fieldwork at one of this organization’s different sites in the same city. |
| 7. | Participant knowledge of the interviewer | What did the participants know about the researcher? e*.g. personal goals, reasons for doing the research* | All participants were briefed on the purpose of the study and also informed that this project was part of the first authors PhD project. He also disclosed that he used to work for the host organization but was no longer affiliated with them in any way. It was conveyed to both interview and focus group participants that the goal of the project was to better understand the ways in which residents and staff experienced and perceived the on-site OPS room. It was also explained that this was part of a PhD project which conducted similar research in Birmingham (UK) and Athens (Greece). |
| 8. | Interviewer characteristics | What characteristics were reported about the interviewer/facilitator? e.g. *Bias, assumptions, reasons and interests in the research topic* | During the community consultation and prior to each interview and focus group, the interviewer discussed his previous research and professional experience in HOPS and OPS as well as his wider interest in better understanding how to improve services and outcomes for people who use drugs. In these discussions, participants were given the opportunity to ask questions relating to the interviewer and the project. |
| Domain 2: Study Design |  |  |  |
| Theoretical Framework |  |  |  |
| 9. | Methodological orientation and Theory | What methodological orientation was stated to underpin the study? *e.g. grounded theory, discourse analysis, ethnography, phenomenology, content analysis* | This study was oriented through a rapid-ethnographic approach. Additionally, although informed by our understanding of the Risk Environment Framework (Rhodes, 2002) and in particular the ways in which environmental, social and policy factors produce risk within housing environments (Braubach & Fairburn, 2010; Ivsins et al., 2022), all codes were developed inductively from the data. |
| Participant Selection |  |  |  |
| 10. | Sampling | How were participants selected? *e.g. purposive, convenience, consecutive, snowball* | See methods section of manuscript. |
| 11. | Method of approach | How were participants approached? e*.g. face-to-face, telephone, mail, email* | Participants were approached face-to-face in the facility. |
| 12. | Sample size | How many participants were in the study? | See methods section of manuscript. |
| 13. | Non-participation | How many people refused to participate or dropped out? Reasons? | 3 people turned down the opportunity of participating in a rapid ethnographic interview, 5 people turned down the opportunity of participating in a focus group and no staff turned down a semi-structured interview. |
| Setting |  |  |  |
| 14. | Setting of data collection | Where was the data collected? e*.g. home, clinic, workplace* | In a secure room within the housing facility. |
| 15. | Presence of non-participants | Was anyone else present besides the participants and researchers? | No, not during any of interviews or focus groups however staff were present during the non-participant observation phase of the fieldwork. |
| 16. | Description of sample | What are the important characteristics of the sample? *e.g. demographic data, date* | See methods section of manuscript. |
| Data Collection |  |  |  |
| 17. | Interview guide | Were questions, prompts, guides provided by the authors? Was it pilot tested? | The question protocols were developed by all of the authors. The protocol was then checked with a resident sample during an initial community consultation. During this meeting residents gave suggestions on appropriate prompts and also suggested additional question (eg., residents suggested that we have a question related to the interactions between residents who use the OPS room and those who don’t). |
| 18. | Repeat interviews | Were repeat interviews carried out? If yes, how many? | No. |
| 19. | Audio/visual recording | Did the research use audio or visual recording to collect the data? | Audio and handwritten notes, see methods section of manuscript. |
| 20. | Field notes | Were field notes made during and/or after the interview or focus group? | Yes, see methods section of manuscript. |
| 21. | Duration | What was the duration of the interviews or focus group? | See methods section of manuscript. |
| 22. | Data saturation | Was data saturation discussed? | Data saturation was discussed by the research team. Originally, we intended to conduct 5 focus groups but felt that along with the interviews, data saturation had been reached and so stopped at 3 focus groups. |
| 23. | Transcripts returned | Were transcripts returned to participants for comment and/or correction? | Focus group and staff interviews were not returned to participants for comment and/or correction however rapid-ethnographic interview notes and quotes were checked for accuracy by all participants following the interview. |
| Domain 3: Analysis and Findings |  |  |  |
| Data Analysis |  |  |  |
| 24. | Number of data coders | How many data coders coded the data? | The lead author led on the coding framework given their role at the site and familiarity with data; however, emerging themes were discussed and amended collaboratively amongst the remaining co-authors. |
| 25. | Description of the coding tree | Did authors provide a description of the coding tree? | Yes, a description of the various codes is included in the main text. |
| 26. | Derivation of themes | Were themes identified in advance or derived from the data? | Themes were derived from the data. |
| 27. | Software | What software, if applicable, was used to manage the data? | Nvivo version 12 software was used to transcribe and code the data and Zotero version 6 was used as a reference management software. |
| 28. | Participant checking | Did participants provide feedback on the findings? | No, participants did not provide feedback on the findings. |
| Reporting |  |  |  |
| 29. | Quotations presented | Were participant quotations presented to illustrate the themes / findings? Was each quotation identified? e*.g. participant number* | Yes. |
| 30. | Data and findings consistent | Was there consistency between the data presented and the findings? | Yes. |
| 31. | Clarity of major themes | Were major themes clearly presented in the findings? | Yes. |
| 32. | Clarity of minor themes | Is there a description of diverse cases or discussion of minor themes? | Not in this manuscript, however a longer form version of this paper will be included in the lead authors doctoral thesis will which contain a broader discussion on the minor themes. |

**Appendix 2: Focus Group Protocol (Residents)**

| *Question* | *Possible Prompt* |
| --- | --- |
| How long have you been staying here? | How did you find out about this facility? |
| How have you found staff here so far? | Are there any characteristics you particularly like or dislike about the facility? Generally, have you felt safe during your stay here? Is there anything in particular which makes you feel more or less safe? |
| Have you stayed in any other similar housing facilities? | If yes, is there anything that is different about this one compared to previous facilities you have stayed in? How have you found being in a housing facility where there are some people who use drugs and some people who do not? |
| What are your opinions of the HOPS room? | What are your opinions of having a HOPS in a facility where some people use drugs and some people don’t? Is the current OPS room effective in supporting ensuring access to the residents who could benefit from it? Do people still ever use drugs in areas of the building or the vicinity of the building which are not the HOPS? Is there anything you would change about the current HOPS? |
| Is there anything that you particularly like or would change about the day-to-day operations of the facility? | How have you found the day-to-day structure of the facility (room opening/laundry/meal/etc times)? |
| How have you found the other services that are on offer here? | How have you found the style and quality of the case planning here? Do you feel like your needs have been met by the case planners? How did you find the intake process? Was there anything in particular which stood out to you during the intake process? |
| What are you views on the provision of harm reduction equipment at the front desk? | Are you happy with the harm reduction equipment that is currently provided? |
| What impact do you think it would have if the HOPS room was closed down? | Who do you think would be most impacted by this? |

**Appendix 3: Rapid-Ethnographic Interview Informal Protocol (Residents)**

| *Question* |
| --- |
| How have you found your stay here so far? |
| What are your views on the HOPS? Is there anything you would change about it? |
| How would you describe the relationship between the staff and residents? |
| How would you describe the relationship between residents who use drugs and those who do not? |
| Is there anything you particularly like or dislike about the day-to-day operations of the facility? |
| Have you felt like your life has changed for better or worse since being a resident here? |

**Appendix 4: Sem-Structured Interview Protocol (Staff)**

| *Question* | *Possible Prompt* |
| --- | --- |
| In your view how does staying here impact the health and well-being of residents? | In your view is harm reduction an important component of that? |
| What are your views on the OPS room? | Do you think it is effective in achieving its aims? |
| Are there any challenges that you face in your day-to-day role? | If yes, what, why and what do you think could be done to alleviate it? |
| In your view how does this housing facility differ to any of the other facilities provided by this organisation? | If yes, what are the key elements? |
| How would you describe the relationship between staff and residents? | Are there things which particularly effect this relationship? |
| How would you describe the relationship between residents who don’t use drugs and those residents who do? | Are there things which particularly effect this relationship? |
| Is there anything that you would change about the how the facility runs? | If yes, what are they and why? |
| Do you believe that there are any major misconceptions that the public has about facilities such as this one? | If yes, what are why? Do you believe that these misconceptions effect residents in any way? |
